# Factors influencing non-adherence to opioids in cancer patients: a mixed-methods cross-sectional study

**DOI:** 10.12688/f1000research.27725.2

**Published:** 2021-03-15

**Authors:** Rattaphol Seangrung, Mallika Ahuja, Koravee Pasutharnchat, Rungwipha Mahawan

**Affiliations:** 1 Department of Anesthesiology, Faculty of Medicine, Ramathibodi Hospital, Mahidol University, Bangkok, 10400, Thailand; 2 Department of Anesthesiology, Maharat Nakhon Ratchasima Hospital, Nakhon Ratchasima, 30000, Thailand

**Keywords:** Opioid, medication non-adherence, cancer, pain management

## Abstract

**Background: **Strong opioids are mainly utilized to attenuate pain in cancer patients. Adherence to analgesic drugs significantly promotes adequate pain management and improves quality of life. ****We aimed to identify the factors influencing non-adherence to strong opioids in cancer patients.

**Methods: **A descriptive, cross-sectional, two-phased, mixed methods design was conducted prospectively to evaluate a cohort of 101 cancer patients who are currently prescribed strong opioids from a pain clinic in Thailand between January and March 2018. ****Participants were asked to complete a questionnaire that included the following sections: general characteristics; the Medication Taking Behavior in Thai (MTB-Thai) for assessing adherence to medications; and factors influencing nonadherence, which were analyzed using multivariate logistic regression. In addition, face-to-face in depth interviews were conducted with patients showing non-adherence to strong opioids (MTB-Thai score ≤21) and analyzed using thematic content analysis.

**Results: **Of 101 cancer pain patients that completed the questionnaire, 39.6% showed non-adherence to strong opioids. Illness understanding (P=0.047) and the use of more than three types of pain medication (P=0.032) were significant factors influencing non-adherence. Qualitative analysis indicated that fear of long-term outcomes, opioid side effects, ineffective pain control, attempts to make the regimen more acceptable, poor understanding, and non-acceptance of disease related to non-adherence.

**Conclusion: **Non-adherence to opioids for cancer patients is a common problem. Awareness of patient factors, medication-related factors, and illness-related factors will provide the knowledge and adequate advice that may enhance adherence to medications.

## Introduction

Cancer is the one of four non-communicable diseases that makes up the majority of global deaths ^1^. In Thailand, an average of 170,000 people were newly diagnosed with cancer in 2018, according to the World Health Organization report ^2^. More than one-third of cancer patients experienced moderate or severe pain ^3^. Improper pain management can be caused by a multitude of factors, including the clinicians’ attitude, patients’ perception, caregiver’s perspective, and the availability or accessibility of analgesic drugs ^4– 9^. Significantly, poor adherence to the analgesic regimen can contribute to ineffective cancer pain management ^10– 12^. Also, it can lead to a substantial worsening of the disease, death, and increased health care costs ^12, 13^.

Strong opioids are the mainstay for treatment of cancer pain. The reported incidence of poor opioid adherence is 50–70% of patients with advanced cancer ^14^. Previous research on the causes of non-adherence has identified various factors, such as illness, drugs, medical personnel, patient characteristics, and socioeconomic factors ^15^. Notably, poor compliance is associated with young age, smoking, fear of drug dependence and side effects, the experience of adverse events, misunderstanding of prescriber instructions, poor beliefs and perceptions, poor family support, and non-acceptance of illness ^7, 16– 20^. However, no study in Thailand has explored the issue of opioid non-adherence in patient-related factors, which is one of the most significant barriers and is a severe problem that obstructs pain management goals. Furthermore, non-adherence to opioids remains a significant health problem, and more high-quality studies are needed to assess these aspects. The study aims to explore the factors influencing opioids non-adherence in cancer patients by using mixed-methods design.

## Methods

The present study was a descriptive, cross-sectional, two-phased mixed methods study using both quantitative and qualitative approaches. The study was conducted between January and March 2018

### Ethical considerations

This study was approved by the Ethics Committee (Chairman Assistant Professor Dr. Chusak Okascharoen) of Faculty of Medicine Ramathibodi Hospital, Mahidol University, Bangkok, Thailand (09-60-05, 11 January 2018). Participants were informed about the study and provided written informed consent to participate in both the questionnaire and the interviews. All data were confidential.

### Participants

The sample size for the study was determined using Taro Yamane sample size formula with 95% confidence level ^21^. The calculation formula of Taro Yamane is presented as: n = N/1 + N (e) ^2^

where: n = sample size required, N = number of people in the population.

In this study cancer pain patients attending a pain clinic at Ramathibodi Hospital, Bangkok, Thailand were the sample population and numbered 134 in the last three months between January and March 2018; therefore, e = allowable error (%)-0.05. A minimum of 101 cancer pain patients who used strong opioids (fentanyl, methadone, morphine) by oral and transdermal routes of administration were required to meet the sample size. Participants who had the follow-up appointment schedule were selected using simple random sampling by computer generated random list. They were approached during a routine follow up at pain clinic.

The inclusion criteria were patients aged 18 years or older, diagnosed with cancer pain, strong opioid analgesics for cancer pain prescribed for more than one week for around-the-clock use or as needed, and ability to communicate well in Thai. Exclusion criteria were patients who declined to participate, and who had known or suspected psychotic disease.

### Questionnaire

All participants filled out the questionnaire by themselves and participated in the interviews at the hospital. The questionnaire assessed demographic characteristics, pain severity (numerical rating scale in the past week and at the moment) with therapeutic outcomes (pain affect working and social activities, routine daily activities and life), and medication adherence using the Medication Taking Behavior in Thai (MTB-Thai) measure (the total score was between 6 and 24, if score ≤21 indicated non-adherence) ^22, 23^. Other factors associated with non-adherence to strong opioids, including patient factors (knowledge of strong opioid analgesics and patient beliefs about strong opioid analgesics) were assessed using a copyrighted Thai version of the self-report Belief about Medication Questionnaire [Thai-BMQ]) ^24, 25^. For the questions about knowledge assessment of strong opioid analgesics were created by pain specialist. Socioeconomic factors (family and social support), medical personnel factors (satisfy and confident of medical service and staffs), medication-related factors (taste, cost, type of medicines, frequency of taking and side effects) and illness-related factors were also assessed. Both MTB-Thai and Thai-BMQ were used in the study with permission of the originators of the questionnaires and completely validated. The full questionnaire used in this study was approved by three pain specialists. Content validity was determined by obtaining the item objective congruence (IOC) value for each questionnaire (including general information and pain severity, which influence non-adherence to opioids, MTB-Thai and Thai-BMQ) ranged from 0.80 to 0.92. All were >0.5, indicating good content validity. Cronbach’s alpha coefficient of the questionnaire ranged from 0.702 to 0.788; a score of >0.7 indicates acceptable internal consistency ^26, 27^. The questionnaire was not modified after the pilot with 10 patients. A copy of the questionnaire can be found in the *Extended data* ^26, 27^.

### In-depth interviews

Open-ended interview questions were included at the end of the questionnaire for patients who had a MTB-Thai score ≤21, in order to provide further commentary and suggest other factors that may influence non-adherence to strong opioids. The open-ended of the interview included four questions related to the experienced of using strong opioids for pain control and outcomes, patients’ concern, and healthcare service system ^26, 27^. These questions were asked in a face-to-face qualitative in-depth interviews by the second (MA) and the fourth author (RM) that were conducted until data saturation was reached. The interviews were audio-recorded with the patients’ permission, and the interviewers also took field notes.

### Statistical analysis

SPSS for Windows version 18.0 (SPSS Inc., Chicago, IL, USA) was used for quantitative data analysis. Descriptive statistics, such as frequencies, percentages, means, and standard deviations, were used to analyze demographic data. Chi-square test, relative risk, 95% confidence intervals (CI), and p-values were used to measure the association between factors and strong opioid analgesic non-adherence. Multiple logistic regression was performed to identify risk factors for opioid non-adherence and to calculate adjusted risk ratios. P-values less than 0.05 were considered statistically significant.

Interview data (transcribed verbatim from recordings) and the interviewers’ memos were subject to thematic content analysis using ATLAS.ti software version 8.0. Using five stages of data analysis in the framework approach included: 1) familiarization or immersion in the raw data to list key ideas and recurrent themes, 2) identifying a thematic framework (all the key issues, concepts, and themes), 3) indexing or applying the thematic framework or index systematically to all the data in textual form by annotating the transcripts with numerical codes, 4) charting or rearranging the data according to the appropriate part of the thematic framework to which they relate, and forming charts, and 5) mapping and interpretation by using the charts to define concepts, map the range and nature of phenomena. 

## Results

### Quantitative survey

**Figure 1.**
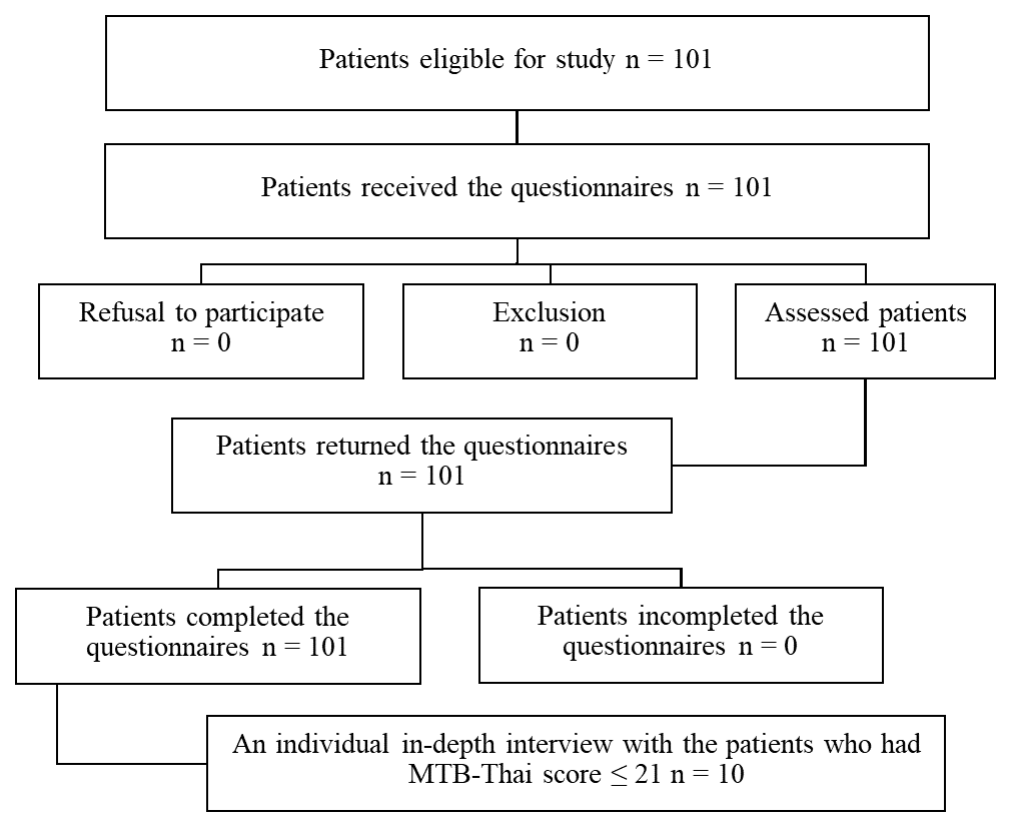
Patient selection flowchart. MTB-Thai score = Medication taking behavior scale in Thai ^22^.

All 101 participants completed the questionnaire ( Figure 1). Most participants were women (N = 52, 51.49%), and the average age was 60.14 years. in total, 40 patients (39.6%) reported non-adherence; the others reported moderate to high adherence. The mean duration of pain treatment was three months (range: 1-11 months). Table 1 shows the demographic data.

**Table 1.** Demographic and clinical characteristics of participants (n = 101).

Characteristics	Data
Gender n (%)	
Male	49 (48.51)
Female	52 (51.49)
Age (years) Mean ± SD	60.14 ± 12.17
Cancer type n (%) Head and Neck Breast Lung Gastrointestinal Genitourinary Hematologic Others	13 (12.87) 15 (14.85) 15 (14.85) 28 (27.72) 13 (12.87) 8 (7.92) 9 (8.91)
Metastatic cancer n (%)	
Metastasis	57 (56.44)
Without metastasis	44 (43.56)
Duration of Pain (months) Median (IQR)	12 (5–15)
Duration of Pain Treatment (months) Median (IQR)	3 (1–11)
Numbers of analgesics n (%)	
>3 ≤3	22 (21.78) 79 (78.22)
Types of analgesics n (%)	
Sustained-release opioids Immediate-release opioids	65 (64.36) 101 (100)
Opioid transdermal patch Anticonvulsants Antidepressants Others	28 (27.72) 69 (68.32) 47 (46.53) 8 (7.92)
Marital Status n (%)	
Single	21 (20.79)
Married	59 (58.42)
Divorced/Separated	8 (7.92)
Widowed	13 (12.87)
Education n (%)	
None	6 (5.94)
Primary school	37 (36.63)
Junior high school	8 (7.92)
Senior high school/Vocational certificate	9 (8.91)
High vocational certificate	8 (7.92)
Bachelor’s degree	23 (22.77)
≥Master’s degree	10 (9.9)
Career n (%)	
Unemployed	40 (39.6)
Student	27 (26.73)
Government employee/State enterprises	3 (2.97)
Company employee	10 (9.9)
Business owner	7 (6.93)
Freelance	6 (5.94)
Others	8 (7.92)
Income (Baht/month) n (%)	
No income	38 (37.62)
<10,000	17 (16.83)
10,001–20,000	13 (12.87)
20,001–30,000	12 (11.88)
30,001–50,000	14 (13.86)
50,001–100,000	6 (5.94)
>100,000	1 (0.99)
Health Scheme n (%)	
Self-pay	12 (11.88)
Universal coverage scheme	41 (40.59)
Social security scheme	5 (4.95)
Government State Enterprise Office scheme	38 (37.62)
Health insurance	1 (0.99)
Others	4 (3.96)
Smoking n (%)	
Never	54 (53.47)
Quit	42 (41.58)
Still smokes	5 (4.95)
Alcohol Drinking n (%)	
Never	52 (51.49)
Quit	47 (46.53)
Still drinks	2 (1.98)

SD = Standard deviation, IQR = Interquartile range.

Statistically significant differences between the adherence and non-adherence groups were found in the general-overuse dimension of the Thai-BMQ (P = 0.047), illness understanding (P = 0.028), and use of more than three types of pain medication (P = 0.035) ( Table 2).

**Table 2.** Factors associated with non-adherence to opioids (MTB-Thai score *^a^* ≤21).

	Non- adherence	Adherence	P-value
Gender n (%)			0.516
Male	21 (52.5)	28 (45.9)	
Female	19 (47.5)	33 (54.1)	
Age (years) Mean ± SD	59.73 ± 11.04	60.41 ± 12.94	0.184
Cancer type n (%)			>0.999
Head and Neck Breast Lung Gastrointestinal Genitourinary Hematologic Others	5 (12.5) 6 (15) 6 (15) 12 (30) 5 (12.5) 3 (7.5) 3 (7.5)	8 (13.11) 9 (14.75) 9 (14.75) 16 (26.23) 8 (13.11) 5 (8.2) 6 (9.84)	
Metastatic cancer n (%) Metastasis Without metastasis	20 (50) 20 (50)	37 (60.66) 24 (39.34)	0.291
Duration of Pain (months) Median (IQR)	11.5 (6–13.5)	12 (5–15)	0.774
Duration of Pain Treatment (months) Median (IQR)	2 (1–7.5)	4 (2–12)	0.188
Numbers of analgesics n (%) >3	13 (32.5)	9 (14.75)	0.035 ^*^
≤3	27 (67.5)	52 (85.25)	
Types of analgesics n (%)			
Sustained-release opioids	25 (62.5)	40 (65.57)	0.752
Immediate-release opioids	40 (100)	61 (100)	-
Opioid transdermal patch	14 (35)	14 (22.95)	0.186
Anticonvulsants	30 (75)	39 (63.93)	0.242
Antidepressants	23 (57.5)	24 (39.34)	0.074
Others	3 (7.5)	5 (8.2)	>0.999
Marital Status n (%)			0.346
Single	6 (15)	15 (24.59)	
Married	25 (62.5)	34 (55.74)	
Divorced/Separated	5 (12.5)	3 (4.92)	
Widowed	4 (10)	9 (14.75)	
Education n (%)			0.371
None	1 (2.5)	5 (8.2)	
Primary school	12 (30)	25 (40.98)	
Junior high school	6 (15)	2 (3.28)	
High vocational certificate	3 (7.5)	5 (8.2)	
Bachelor’s degree	10 (25)	13 (21.31)	
≥Master’s degree	4 (10)	6 (9.84)	
Career n (%)			0.579
Unemployed	15 (37.5)	25 (40.98)	
Student	14 (35)	13 (21.31)	
Government employee/State enterprises	1 (2.5)	2 (3.28)	
Company employee	4 (10)	6 (9.84)	
Business owner	2 (5)	5 (8.2)	
Freelance	3 (7.5)	3 (4.92)	
Others	1 (2.5)	7 (11.48)	
Income (Baht/month) n (%)			0.14
No income	14 (35)	24 (39.34)	
<10,000	6 (15%)	11 (18.03)	
10,001–20,000	7 (17.5%)	6 (9.84)	
20,001–30,000	7 (17.5)	5 (8.2)	
30,001–50,000	2 (5)	12 (19.67)	
50,001–100,000	4 (10)	2 (3.28)	
>100,000	0 (0)	1 (1.64)	
Health Scheme n (%)			0.847
Self-pay	6 (15)	6 (9.84)	
Universal coverage scheme	15 (37.5)	26 (42.62)	
Social Security Scheme	1 (2.5)	4 (6.56)	
Government of State Enterprise Officer scheme	16 (40)	22 (36.07)	
Health insurance	0 (0)	1 (1.64)	
Others	2 (5)	2 (3.28)	
Smoking n (%)			0.205
Never	18 (45)	36 (59.02)	
Quit	21 (52.5)	21 (34.43)	
Still smokes	1 (2.5)	4 (6.56)	
Alcohol Drinking n (%)			0.712
Never	18 (45)	34 (55.74)	
Quit	21 (52.5)	26 (42.62)	
Still drinks	1 (2.5)	1 (1.64)	
Average pain score last week (0–10)			
Median (IQR)	7 (5–9)	6 (4–8)	0.058
Mean ± SD	6.75 ± 2.46	5.57 ± 2.76	
Average pain score now (0–10)			
Median (IQR)	5 (3.5–7)	5 (3–6)	0.196
Mean ± SD	5.38 ± 2.68	4.62 ± 2.75	
Effect on work and social life n (%)	36 (90)	50 (81.97)	0.267
Effect on daily routine n (%)	35 (87.5)	51 (83.61)	0.59
Overall effect of pain on life n (%)			0.444
Not at all	0 (0)	4 (6.56)	
Little impact	3 (7.5)	5 (8.2)	
Moderate impact	9 (22.5)	15 (24.59)	
Large impact	16 (40)	17 (27.87)	
Extremely large impact	12 (30)	20 (32.79)	
***Patient Factors*** Knowledge Score (0–10)			
Median (IQR)	9 (8–9)	8 (7–9)	0.788
Mean ± SD	8.13 ± 1.56	8.16 ± 1.4	
Belief about Medication (Thai-BMQ *^b^*)			
Specific-Necessity (5–25)			
Median (IQR)	20 (17.5–23)	20 (19–22)	0.514
Mean ± SD	19.85 ± 3.66	20.31 ± 2.57	
Specific-Concern (5–25) *reversed ^c^*			
Median (IQR)	14 (11–17.5)	16 (12–19)	0.17
Mean ± SD	14.38 ± 4.61	15.67 ± 4.62	
General-Overuse (4–20) *reversed ^c^*			
Median (IQR)	11 (10–12)	12 (11–13)	0.047 ^*^
Mean ± SD	11.05 ± 2.63	11.97 ± 2.43	
General-Harm (4–20) *reversed ^c^*			
Median (IQR)	12 (10.5–15.5)	13 (12–14)	0.806
Mean ± SD	12.33 ± 3.28	12.54 ± 2.27	
***Family and social support*** Caregiver n (%)			0.898
Self-care	19 (47.5)	29 (47.54)	
Relatives	18 (45)	29 (47.54)	
Non-relatives	3 (7.5)	3 (4.92)	
Help provided if needed n (%)			0.382
No	3 (7.5)	2 (3.28)	
Yes	37 (92.5)	59 (96.72)	
Satisfaction with pain clinic n (%)			0.789
Satisfied	6 (15)	8 (13.11)	
Very satisfied	34 (85)	53 (86.89)	
Confidence in pain clinic n (%)			>0.999
Not sure	0 (0)	1 (1.64)	
Confident	40 (100)	60 (98.36)	
***Medication-related factors*** n (%)			
Drugs taste bad	6 (15)	16 (26.23)	0.181
Drugs too expensive	7 (17.5)	17 (27.87)	0.231
Too many types of drugs	6 (15)	11 (18.03)	0.690
Need to take drugs too often	5 (12.5)	7 (11.48)	>0.999
Too many side effects	11 (27.5)	9 (14.75s)	0.207
***Illness understanding and acceptance***			
Median (IQR)	4 (3–4)	4 (4–4)	0.028 ^*^
Mean ± SD	3.48 ± 0.75	3.72 ± 0.64	

SD = Standard deviation, IQR = Interquartile range. ^*a*^MTB-Thai score = Medication taking behavior scale in Thai ^22^. ^*b*^Thai-BMQ = Belief about Medication Questionnaire, Thai version ^24^. ^*c*^Reversed scale. *P < 0.05 (statistically significant).

The multivariate analysis showed that two variables had significant associations with opioid non-adherence: illness understanding (P = 0.047) and use of more than three types of pain medication (P = 0.032). The illness understanding and acceptance part of the questionnaire contained five statements. Most participants agreed with either “I understand my illness, and I think I received the best treatment” or “I understand my illness, but I think I could have received better treatment.” Patients who chose the former response were less likely to show non-adherence than those who chose the latter answer (RR = 0.53, 95% CI [0.283–0.993]). Participants prescribed more than three types of analgesics had a 3.04 times higher risk of medication non-adherence than participants prescribed three or fewer types of medication (RR = 3.04, 95% CI [1.099–8.411]) ( Table 3).

**Table 3.** Univariate and multivariate logistic regression results.

	Non-adherence (n = 40)	Adherence (n = 61)	RR (95% CI)	P-value ^#^	Adjusted RR (95% CI)	P-value
General-Overuse, *reversed ^a^* Median (IQR)	11 (10–12)	12 (11–13)	0.86 (0.725–1.019)	0.082	0.872 (0.729–1.044)	0.136
Illness understanding and acceptance Median (IQR)	4 (3–4)	4 (4–4)	0.597 (0.332–1.075)	0.086	0.53 (0.283–0.993)	0.047 *
Number of drugs >3 n (%)	13 (32.5%)	9 (14.75%)	2.782 (1.056–7.329)	0.038 *	3.04 (1.099–8.411)	0.032 *

IQR = Interquartile range, CI = Confidence interval, RR = Risk ratio. *P < 0.05 (statistically significant). ^#^Results from binary logistic regression analysis (unlike the output from chi-square test in Table 2) ^*a*^Reversed scale.

### Qualitative results

Saturated data, in total 10 individual in-depth interviews with patients who had MTB-Thai score ≤21 were conducted, which lasted around 30 to 45 minutes. Five themes related to opioid non-adherence emerged from the data: fear of long-term outcomes, desirable or undesirable opioid side effects, ineffective pain control, attempts to make the regimen more acceptable, and poor understanding and non-acceptance of the disease. Analytic results of the contextual factors associated with non-adherence to opioids in cancer patients are presented in Table 4.

**Table 4.** Contextual factors associated with non-adherence to opioids in cancer patients.

Contextual factors	Participants mention (N=10): n (%)
***Patient factors: fear of long-term opioid adverse events***	
concern about opioid addiction	4 (40)
concern about opioid and other medication induced organ failure	8 (80)
***Medication-related factors***:***desirable or undesirable opioid side effects***	
concern about opioid-induced constipation	6 (60)
concern about opioid-induced nausea	3 (30)
concern about opioid-induced sedation	4 (40)
***Medication-related factors: ineffective pain control***	
opioids are unable to control the pain sufficiently	4 (40)
***Medication-related factors: attempts to make the regimen more acceptable***	
applied opioids regimen to suit their lifestyle	3 (30)
***Illness-related factors: poor understanding and non-acceptance of the disease***	
discontinue opioids after better pain relief	7 (70)

***Patient factors***

Theme 1: Fear of long-term opioid adverse events

Almost half of the patients were concerned about opioid addiction. Some chose to be in pain to minimize the chance of addiction. As two patients remarked:

          “I could get addicted to the medication, so I don’t want to take morphine syrup more than once a day.”

          “I feel uncomfortable taking morphine in front of people. They look at me like I am addicted to drugs.”

Many patients were afraid of liver or kidney damage after long-term opioid use and combined with other pain medications, despite their physicians confirming the safety of their opioid dosage. As some patients mentioned:

          “Morphine could reduce my pain but if I take as much as I need, I will suffer from liver or kidney disease in the future.”

          “I took lots of medication. I think my liver has had to work too hard, so I wait until I had severe pain, I will take morphine.”

          “These drugs can relief the pain, but I wondered if just one or two drugs could control everything. I think that taking four types of drugs every day will damage my health.”

***Medication-related factors***

Theme 1: Desirable or undesirable opioid side effects

Many patients reported opioid side effects. Some found that the side effects were unbearable and affected their quality of life. Commonly reported severe constipation, upset stomach, and drowsiness. As some patients said:

          “I am in pain but I chose to use as low a dose of morphine as I could because it made me constipated.”

          “Severe nausea made me afraid to use morphine. Throwing up was much worse than living with this pain.”

          “I always had to take a nap after taking morphine. So I could only take it before bedtime otherwise I might sleep all day.”

Notably, some participants found specific side effects beneficial, whereas others found them problematic. As one patient said:

          “The rescue drug makes me sleep well at night. I take it every night even if the pain does not bother me much.”

Theme 2: Ineffective pain control

Some patients do not adhere to medical regimens because they find pain medication ineffective. Some participants reported not taking opioids because they did not have the intended effect. As one patient remarked:

          “I suffered from pain, but the drug did not make me feel as good as I expected. So there was no reason for taking it.” 

Some patients increased their basal opioid doses because their pain had worsened and dared not to call to consult the doctor about ineffective pain control. As one patient remarked:

          “I did not know what to do with my pain anymore. I felt so hopeless. I took morphine syrup every hour. The effect was too short-lived, so I tried to take more morphine tablets than the doctor had prescribed, but I ended up feeling sleepier all day.”

Theme 3: Attempts to make the regimen more acceptable

Some patients applied their medication regimen to suit their lifestyle and drug reactions. Some reduced the medication frequency, as they felt uncomfortable taking medicine at midday. Others changed the regimen from around the clock to after meals, because it was easier to remember. Some took all their drugs at bedtime rather than in the morning because of the sedative effect. As some patients said:

          “I go to work every day. It’s not easy to bring the drugs with me, so I changed the schedule from a three times daily regimen to a two times daily regimen. I think this work for me.”

          “I take the morphine after meals. It is easier to remember. I used to forget to take the pills at 2 pm, which made the pain worse in the evening.”

          “I felt sleepy after I took the pills, so I took it all at night before bedtime. This meant that the pain wasn’t well controlled, but that’s better than feeling drowsy all day.”

***Illness-related factors***

Theme 1: Poor understanding and non-acceptance of the disease

If doctors can improve patients’ understanding and acceptance of illness, then more collaborative treatment decisions can be made. If patients insist on seeking complete pain relief or a complete cure and are reluctant to adopt modified life goals and activities, it is difficult to set realistic treatment goals. Acceptance-based pain management may be helpful for cancer patients. Many patients reduced or discontinued opioids by themselves when their pain was decreasing. As two patients said:

          “I took the pills everyday as advised and they controlled the pain well. I wondered if I could stop taking these drugs, so I tried to stop and the pain came back. Are these drugs helping me with the disease? Or I should try something else?”

          “After taking the drug, I feel better, I stopped taking it on some day. I think the disease is getting better.”

## Discussion

The main purpose of this mixed-methods study design was to explore the factors influencing non-adherence to strong opioids in cancer pain patients. Most research has studied the clinical factors associated with adherence to opioids for cancer pain and has not focused the medical non-adherence factor alone. Investigation of non-adherence to opioids in cancer patients is a pro-active useful model for correcting beliefs, attitudes and behavior. This study asked participants about characteristics related to themselves, family and social support, doctor-patient relationships, medication-related factors, and illness-related factors. The incidence of non-adherence of our study was 39.6%, similar to previous study ^28^. Our data indicate that only three out of these five factors were significantly associated with non-adherence: patient factors, medication-related factors, and illness-related factors.

### Patient factors

In the quantitative data, patients’ knowledge of strong opioid analgesics and their beliefs about medication were not significantly related to non-adherence. However, the interviews revealed a concern about adverse effects, side effects, opioid addiction and multiple organ failure from long-term usage, as reported in previous work ^29– 33^. This was even though most of the patients from this study had a higher education and good knowledge of strong opioids. They may also be concerns about the adverse effects of opioids. Similarly another study found that some patients who were educated about the side effects of medication showed increased concerns about the risk of addiction ^34, 35^.

Generally, cancer patients require long-term use of strong opioids. Physicians should educate patients during treatment about the prevention of adverse effects. Although patient education is a key component of adherence, more education may make patients anxious and fearful about opioid adverse effects. Patient beliefs and attitudes regarding the effectiveness of the treatment, and lack of motivation, also affects medication adherence ^36^. Healthcare providers should reassure patients by emphasizing the benefits rather than the risks of opioids, and should identify patients’ concerns. Additionally, involvement of patients in the treatment decision-making process may help to reduce fear and facilitate adherence ^12, 37^.

### Family and social support

A previous study ^38^ found an association between medical adherence and family and social support: adherence was 1.74 times higher in patients from cohesive families and 1.53 times lower in patients from families in conflict. The present findings did not show a correlation between adherence and family support. More than 90% of patients in both the adherence and non-adherence groups confirmed that they received the best care from their families when needed. This may be because Thai people live in large families and therefore find it relatively easy to obtain assistance when needed.

### Doctor-patient relationships

Almost all patients expressed high satisfaction and confidence in the pain clinic services and staff. Therefore, the doctor-patient relationship was not significantly associated with non-adherence. One previous study ^39^ found a correlation; patients who believed that doctors treat patients as equals, who felt that doctors discuss treatments with patients before making decisions, and who could choose their doctor were more likely to adhere to recommendations.

### Medication-related factors

Both the quantitative and qualitative data revealed many medication-related reasons for non-adherence, which reflected previous study findings ^17, 40, 41^. Our quantitative analysis showed that use of more than three types of drugs was associated with a 3 times higher risk of non-adherence than use of less than three types. Furthermore, the qualitative analysis identified three themes related to medication-related non-adherence factors. Similar to a previous study ^15, 40^, we found that some patients reduced their opioid dose to avoid unbearable side effects, such as constipation, nausea, vomiting, dyspepsia, or drowsiness. Despite having poor pain control, they could not take the opioid dose prescribed because of the side effects. Most said that their pain was much more bearable than the side effects. Some did not inform their doctor about their pain owing to limited time, being considerate or shame. Moreover, some patients used opioids for the wrong reasons; for example, taking opioids at night to help them sleep even when they had no pain.

In accordance with previous findings ^40, 41^, we found that ineffective pain control was a key reason why some patients refused to take prescribed medication. Some patients increased the prescribed opioid dose for maximal pain relief. Some obtained opioids from many different hospitals to control the pain without discussing this with their doctor. Patients reported many reasons for non-adherence. Some felt that their doctor had insufficient time to listen to their problems. Others were afraid that the doctor would abandon them because they did not use the drugs as prescribed.

One of the medication-related factors we identified was the attempt to make the drug regimen more acceptable; this has also been reported in previous work ^40^. Patients changed the drug schedule to suit their lifestyle. Some patients reduced the dose frequency as they were not comfortable taking drugs at certain times of the day. Some changed the interval from around the clock to after meals, as it was easier to remember. Some patients took all their medications at bedtime rather than in the morning because of the sedative effects.

### Illness-related factors

Previous studies of patients with chronic nonmalignant pain indicate that illness acceptance predicts increased psychological, social, and physical functioning ^42, 43^ and that acceptance-based pain management may be helpful for cancer patients ^44^. The present quantitative and qualitative data show that patients with poor illness understanding and non-acceptance of the disease show medication non-adherence. In our study, patients misunderstood their situation and believed that they could be fully cured and become pain free. Some patients believed that there were better treatment options than the treatment they had received. The results suggest that enhancing the acceptance of pain and cancer may be a clinically relevant management goal.

Although many factors were not statistically significant in the quantitative study, such as the patient’s belief about opioid side effects or adverse effects; however, more than three types of pain medication may be the critical factor of non-adherence that might raise their concern about long term adverse effects as opioid addiction or multiple organs failure. Also, taking multiple drugs may be a factor that causes patients to adjust their medication as appropriate, which causes inadequate pain relief. From the in-depth interviews, it was clear that concerns about medication side effects, fear of adverse events and poor pain control were barriers for opioid use in cancer pain management.

### Recommendations

Mainly, the various opioids are very different in bioavailability, metabolism, and response between individual patients. Appropriate opioid use must be selected for each cancer patient, and the dose must be individually titrated. Effective and safe titration of opioids has a significant impact on patient comfort. Obviously, several complex factors affect opioids non-adherence in cancer patients. Therefore, we recommend the following strategies to improve adherence to strong opioid medication for cancer pain.

1. Understanding patients’ reasons for non-adherence to opioids could help doctors to identify how these patients may present clinically, address patients’ concerns about opioids, and encourage doctors to offer patients alternatives to opioid treatment.
2. Reviewing the number of medications because of drug interactions can be managed by reviewing the patient’s medication profile for duplicate or unnecessary medications.
3. Good patient-doctor communication may reduce anxiety, and also improves pain control ^45^. For example, discussing a patient’s concerns about the risk of addiction may help the patient and doctor to set up plans to monitor misuse or identify less risky or more acceptable alternative pain management strategies.

### Limitations

We only measured non-adherence to strong opioid analgesic medicine. The patients might have been taking other medicines for pain control prescribed simultaneously. As these medicines could have affected pain control, they might have confounded the present results.

## Conclusion

Almost half of cancer pain patients prescribed opioids showed non-adherence to the medical regimen. Three factors were significantly associated with medication non-adherence: patient factors (fear of long-term outcomes), medication-related factors (use of more than three types of drugs, side effects, ineffective pain control, attempts to make the regimen more acceptable), and illness-related factors (poor illness understanding and non-acceptance of the disease).

## Data availability

### Underlying data

The recordings and transcription of interviews are not openly available in order to conserve the confidential information of participants. All document files were eradicated immediately following data analysis. Themes and quotes from the data analysis are available in Thai. This data can be obtained by application to the Ethical Committee of Faculty of Medicine Ramathibodi Hospital. To apply, please contact the corresponding author at rattaphol_nu@hotmail.com, who will facilitate this process.

Figshare: Data of factors influencing non-adherence to opioids in cancer patients.xls., https://doi.org/10.6084/m9.figshare.13336691.v2 ^46^.

### Extended data

Figshare: Questionnaire and open ended questions in English.doc., https://doi.org/10.6084/m9.figshare.13336754.v1 ^26^.

Figshare: Thai version of the questionnaire and open ended questions.docx., https://doi.org/10.6084/m9.figshare.13336766.v2 ^27^.

### Reporting guidelines

Figshare: STROBE checklist for ‘Factors influencing non-adherence to opioids in cancer patients: a mixed-methods cross-sectional study’, https://doi.org/10.6084/m9.figshare.13336778.v1 ^47^.

Figshare: COREQ checklist for ‘Factors influencing non-adherence to opioids in cancer patients: a mixed-methods cross-sectional study’, https://doi.org/10.6084/m9.figshare.13336793.v1 ^48^.

Data are available under the terms of the Creative Commons Attribution 4.0 International license (CC-BY 4.0).

## References

[1] World Health Organization: Global Health Observatory. Geneva: World Health Organization;2018; Accessed June 21, 2018. Reference Source

[2] Bray F Ferlay J Soerjomataram I: Global cancer statistics 2018: GLOBOCAN estimates of incidence and mortality worldwide for 36 cancers in 185 countries. *CA Cancer J Clin.* 2018;68(6):394–424. 10.3322/caac.21492 30207593

[3] van den Beuken-van Everdingen MHJ de Rijke JM Kessels AG: Prevalence of pain in patients with cancer: a systematic review of the past 40 years. *Ann Oncol.* 2007;18(9):1437–49. 10.1093/annonc/mdm056 17355955

[4] Jamison RN Sheehan KA Scanlan E: Beliefs and attitudes about opioid prescribing and chronic pain management: survey of primary care providers. *J Opioid Manag.* 2014;10(6):375–82. 10.5055/jom.2014.0234 25531955

[5] Potter VT Wiseman CE Dunn SM: Patient barriers to optimal cancer pain control. *Psychooncology.* 2003;12(2):153–60. 10.1002/pon.627 12619147

[6] Liang SY Yates P Edwards H: Factors influencing opioid-taking self-efficacy and analgesic adherence in Taiwanese outpatients with cancer. *Psychooncology.* 2008;17(11):1100–07. 10.1002/pon.1326 18314911

[7] Gunnarsdottir S Donovan HS Serlin RC: Patient-related barriers to pain management: the Barriers Questionnaire II (BQ-II). *Pain.* 2002;99(3):385–96. 10.1016/s0304-3959(02)00243-9 12406513

[8] Lee BO Liu Y Wang YH: Mediating Effect of Family Caregivers' Hesitancy to Use Analgesics on Homecare Cancer Patients' Analgesic Adherence. *J Pain Symptom Manag.* 2015;50(6):814–21. 10.1016/j.jpainsymman.2015.06.014 26297852

[9] Reid CM Gooberman-Hill R Hanks GW: Opioid analgesics for cancer pain: symptom control for the living or comfort for the dying? A qualitative study to investigate the factors influencing the decision to accept morphine for pain caused by cancer. *Ann Oncol.* 2008;19(1):44–8. 10.1093/annonc/mdm462 18073222

[10] Miaskowski C Dodd MJ West C: Lack of adherence with the analgesic regimen: a significant barrier to effective cancer pain management. *J Clin Oncol.* 2001;19(23):4275–79. 10.1200/JCO.2001.19.23.4275 11731509

[11] Valeberg BT Miaskowski C Hanestad BR: Prevalence rates for and predictors of self-reported adherence of oncology outpatients with analgesic medications. *Clin J Pain.* 2008;24(7):627–36. 10.1097/AJP.0b013e31816fe020 18716502

[12] Osterberg L Blaschke T: Adherence to medication. *N Engl J Med.* 2005;353(5):487–97. 10.1056/NEJMra050100 16079372

[13] Cutler RL FernandezLlimos F Frommer M: Economic impact of medication non-adherence by disease groups: a systematic review. *BMJ Open.* 2018;8(1):e016982. 10.1136/bmjopen-2017-016982 29358417 PMC5780689

[14] Abbas SQ Abbas Z: Is opiate compliance a problem in cancer pain? A survey of health-care professionals' views. *Int J Palliat Nurs.* 2003;9(2):56–63. 10.12968/ijpn.2003.9.2.56 12668940

[15] Pound P Britten N Morgan M: Resisting medicines: a synthesis of qualitative studies of medicine taking. *Soc Sci Med.* 2005;61(1):133–55. 10.1016/j.socscimed.2004.11.063 15847968

[16] Jin J Sklar GE Min Sen Oh V: Factors affecting therapeutic compliance: A review from the patient's perspective. *Ther Clin Risk Manag.* 2008;4(1):269–86. 10.2147/tcrm.s1458 18728716 PMC2503662

[17] Ingersoll KS Cohen J: The impact of medication regimen factors on adherence to chronic treatment: a review of literature. *J Behav Med.* 2008;31(3):213–24. 10.1007/s10865-007-9147-y 18202907 PMC2868342

[18] Alhewiti A: Adherence to Long-Term Therapies and Beliefs about Medications. *Int J Family Med.* 2014;2014:479596. 10.1155/2014/479596 24688792 PMC3943193

[19] Kleinsinger F: The Unmet Challenge of Medication Nonadherence. *Perm J.* 2018;22:18–033. 10.7812/TPP/18-033 30005722 PMC6045499

[20] Nguyen LMT Rhondali W De la Cruz M: Frequency and predictors of patient deviation from prescribed opioids and barriers to opioid pain management in patients with advanced cancer. *J Pain Symptom Manage.* 2013;45(3):506–16. 10.1016/j.jpainsymman.2012.02.023 22940562 PMC3856203

[21] Yamane T: Statistics, An Introductory Analysis. Harper and Row, New York, NY, USA, 2 ^nd^edition,1967. Reference Source

[22] Sakthong P Sonsa-Ardjit N Sukarnjanaset P: Development and psychometric testing of the medication taking behavior tool in Thai patients. *Int J Clin Pharm.* 2016;38(2):438–45. 10.1007/s11096-016-0275-8 26942440

[23] Sooksai N Rittirod T Pachanasoontorn N: The Guardian’s Beliefs on Psychotropic Medications Adherence of Autistic Pediatric Patients. *Srinagarind Med J.* 2019;34(6):595–601. Reference Source

[24] Leaungsomnapa Y Leaungsomnapa S Promproh S: Confirmatory factor analysis of beliefs about medicine questionnaire in Thai version. *J Prapokklao Hosp Clin Med Educat Center.* 2014;31:297–310.

[25] Sriwarakorn S Krittiyanunt S Sakulbumrungsil R: Sensitivity and Specificity of Thai-Version Brief Medication Questionnaire. *J Health Res.* 2018;24(3):129–34. Reference Source

[26] Seangrung R: Questionnaire and open ended questions in English.doc. *figshare.*Dataset. 10.6084/m9.figshare.13336754.v1

[27] Seangrung R: Thai version of the questionnaire and open ended questions.docx. *figshare.*Dataset.2020. 10.6084/m9.figshare.13336766.v2

[28] Chou WC Chen JS Hung CY: A nationwide survey of adherence to analgesic drugs among cancer patients in Taiwan: prevalence, determinants, and impact on quality of life. *Support Care Cancer.* 2019;27(8):2857–67. 10.1007/s00520-018-4599-x 30552596

[29] Tzeng JI Chang CC Chang HJ: Assessing analgesic regimen adherence with the Morisky Medication Adherence Measure for Taiwanese patients with cancer pain. *J Pain Symptom Manage.* 2008;36(2):157–66. 10.1016/j.jpainsymman.2007.10.015 18411015

[30] Berry PE Ward SE: Barriers to pain management in hospice: a study of family caregivers. *Hosp J.* 1995;10(4):19–33. 10.1080/0742-969x.1995.11882805 8698298

[31] Cherny NI Portenoy RK: The management of cancer pain. *CA Cancer J Clin.* 1994;44(5):262–303. 10.3322/canjclin.44.5.263 8076244

[32] Ho RC: Pain in the cancer patient. *CA Cancer J Clin.* 1994;44(5):259–61. 10.3322/canjclin.44.5.259 8076243

[33] Ward SE Hernandez L: Patient-related barriers to management of cancer pain in Puerto Rico. *Pain.* 1994;58(2):233–8. 10.1016/0304-3959(94)90203-8 7816490

[34] Davis MP Walsh D: Epidemiology of cancer pain and factors influencing poor pain control. *Am J Hosp Palliat Med.* 2004;21(2):137–42. 10.1177/104990910402100213 15055515

[35] Sun VC Borneman T Ferrell B: Overcoming barriers to cancer pain management: an institutional change model. *J Pain Symptom Manage.* 2007;34(4):359–69. 10.1016/j.jpainsymman.2006.12.011 17616336 PMC2747495

[36] Horne R Chapman SC Parham R: Understanding patients' adherence-related beliefs about medicines prescribed for long-term conditions: a meta-analytic review of the Necessity-Concerns Framework. *PLoS One.* 2013;8(12):e80663. 10.1371/journal.pone.0080633 24312488 PMC3846635

[37] Haynes RB McDonald HP Garg AX: Helping patients follow prescribed treatment: clinical applications. *JAMA.* 2002;288(22):2880–3. 10.1001/jama.288.22.2880 12472330

[38] DiMatteo MR: Social support and patient adherence to medical treatment: a meta-analysis. *Health Psychol.* 2004;23(2):207–18. 10.1037/0278-6133.23.2.207 15008666

[39] Stavropoulou C: Non-adherence to medication and doctor-patient relationship: Evidence from a European survey. *Patient Educ Couns.* 2011;83(1):7–13. 10.1016/j.pec.2010.04.039 20541884

[40] Lewis ET Combs A Trafton JA: Reasons for under-use of prescribed opioid medications by patients in pain. *Pain Med.* 2010;11(6):861–71. 10.1111/j.1526-4637.2010.00868.x 20624241

[41] Crosby FE Colestro J Ventura MR: Survey of pain among veterans in Western New York. *Pain Manag Nurs.* 2006;7(1):12–22. 10.1016/j.pmn.2005.12.001 16490732

[42] McCracken LM Eccleston C: A prospective study of acceptance of pain and patient functioning with chronic pain. *Pain.* 2005;118(1–2):164–9. 10.1016/j.pain.2005.08.015 16203093

[43] McCracken LM Spertus IL Janeck AS: Behavioral dimensions of adjustment in persons with chronic pain: pain-related anxiety and acceptance. *Pain.* 1999;80(1–2):283–9. 10.1016/s0304-3959(98)00219-x 10204741

[44] Xu X Cheng Q Ou M: Pain acceptance in cancer patients with chronic pain in Hunan, China: A qualitative study. *Int J Nurs Sci.* 2019;6(4):385–91. 10.1016/j.ijnss.2019.09.011 31728390 PMC6838986

[45] Banja JD: Empathy in the physician's pain practice: benefits, barriers, and recommendations. *Pain Med.* 2006;7(3):265–75. 10.1111/j.1526-4637.2006.00159.x 16712628

[46] Seangrung R: Data of factors influencing non-adherence to opioids in cancer patients.xls. *figshare.*Dataset.2020. 10.6084/m9.figshare.13336691.v2 PMC7984143 33815776

[47] Seangrung R: STROBE checklist cross-sectional study of factors influencing non-adherence to opioids in cancer patients.docx. *figshare.*Dataset. 10.6084/m9.figshare.13336778.v1 PMC7984143 33815776

[48] Seangrung R: COREQ Checklist for factors influencing non-adherence to opioids in cancer patients.pdf. *figshare.*Dataset. 10.6084/m9.figshare.13336793.v1 PMC7984143 33815776

