# Supplementary material for: Factors influencing non-adherence to opioids in cancer patients: a mixed-methods cross-sectional study
Source: F1000Res. 2021 Mar 15;9:1471. Originally published 2020 Dec 16. [Version 2] doi: 10.12688/f1000research.27725.2 (PMC7984143; doi:10.12688/f1000research.27725.2)
Supplement: Peer review history

# Peer review: Reviewer response for version 2

**DOI:** 10.5256/f1000research.55051.r81445

**Published:** 2021-03-19

**Authors:** Noraida Mohamed Shah

**Affiliations:** 1 Centre of Quality Management of Medicines, Faculty of Pharmacy, Universiti Kebangsaan Malaysia, Kuala Lumpur, Malaysia

Thank you for the opportunity to review the revised article. I have no further comments for this paper.

Is the work clearly and accurately presented and does it cite the current literature?

Yes

If applicable, is the statistical analysis and its interpretation appropriate?

Yes

Are all the source data underlying the results available to ensure full reproducibility?

Partly

Is the study design appropriate and is the work technically sound?

Yes

Are the conclusions drawn adequately supported by the results?

Yes

Are sufficient details of methods and analysis provided to allow replication by others?

Partly

Reviewer Expertise:

Clinical Pharmacy, medication adherence

I confirm that I have read this submission and believe that I have an appropriate level of expertise to confirm that it is of an acceptable scientific standard.

# Peer review: Reviewer response for version 1

**DOI:** 10.5256/f1000research.30656.r76208

**Published:** 2021-01-11

**Authors:** Noraida Mohamed Shah

**Affiliations:** 1 Centre of Quality Management of Medicines, Faculty of Pharmacy, Universiti Kebangsaan Malaysia, Kuala Lumpur, Malaysia

Thank you for the opportunity to review this article. It is an interesting read. Non-adherence to opioids for cancer pain is well known in the literature. However, the mixed method employed in this study further explore reasons behind non-adherence specific to the Thai population.

 Suggestions/comments related to the article are as follows:

1. English editing is required for this article.
2. Introduction: *“A mixed-methods study is required to study the effects of compliance in enhancing the impact of treatment” *– this sentence is unclear. Was this investigated in this study?

**Method**

1. Please explain simple random sampling used in this study.
2. Please state if Medication Taking Behavior in Thai (MTB-Thai) and Belief about Medication Questionnaire (Thai-BMQ) are validated questionnaires.
3. How were these questionnaires scored? E.g. How did you classify patients as non-adherent based on MTB-Thai? Similarly, for the other questionnaires. 
4. *“item objective congruence (IOC) value for each questionnaire…ranged from 0.80 to 0.92. All were <0.5, indicating good content validity.”* - Please check if this statement is correct.
5. Cite references used for values indicating good content validity and acceptable internal consistency as stated in the method section.

**Results**

1. Would the result be different if number of painkillers is analysed as a continuous variable rather than categorical (< 3 and ≥ 3)? (Table 2).
2. Under Table 2, for Patient Factors, Knowledge score (0-10), which questionnaire was used to assess this? Please elaborate under methods section.
3. Why only 10 patients interviewed for qualitative analysis? What were the questions asked in the interviews (can explain this under method section: in depth interviews).
4. Was the patient who had a desirable opioid effect ( *“The drug makes me sleep well at night. I take it every night even if the pain does not bother me much”*) non-adherent to the pain killer? This seems strange as patient would take the medication if it is producing the desired effects.

**Discussion**

1. Under medication-related factors, it was stated that *“Some did not inform their doctor about their pain owing to limited time, being considerate or shame”*. However, this was not stated under results section. Please clarify.
2. For the statement under illness-related factors: *“Sometimes, patients misunderstood their situation and believed that they could be fully cured and become pain free. Some patients believed that there were better treatment options than the treatment they had received.”* Are these based on your assumption or from the literature?
3. *“however, more than three types of pain medication was the critical factor of non-adherence that might raise their concern about long term adverse effects.”* – how did you relate taking > 3 types of pain medication with concern about long term side effects? Was this shown in your study? Similarly, for the next statement *“Also, taking multiple drugs may be a factor that causes patients to adjust their medication as appropriate, which causes inadequate pain relief”.* How did you relate taking multiple drugs with medication adjustment? Was this seen in your study?

Is the work clearly and accurately presented and does it cite the current literature?

Yes

If applicable, is the statistical analysis and its interpretation appropriate?

Yes

Are all the source data underlying the results available to ensure full reproducibility?

Partly

Is the study design appropriate and is the work technically sound?

Yes

Are the conclusions drawn adequately supported by the results?

Yes

Are sufficient details of methods and analysis provided to allow replication by others?

Partly

Reviewer Expertise:

Clinical Pharmacy, medication adherence

I confirm that I have read this submission and believe that I have an appropriate level of expertise to confirm that it is of an acceptable scientific standard, however I have significant reservations, as outlined above.

# Response

**Published:** 2021-03-06

**Authors:** Rattaphol Seangrung

**Affiliations:** Ramathibodi Hospital, Thailand

Thank you, the reviewer, for your thoughtful suggestions. I have edited the last version under your comments as follows:

**Introduction: **It was written clearly about the sentence of the aims of the study.

**Method:**

1. The sampling method was simple random by a computer-generated random list of the cancer patients who had the follow-up appointment schedule in the study periods.
2. Already state that MTB-Thai and Thai-BMQ are completely validated.
3. If the total score of MTB-Thai ≤21 indicated medical non-adherence, as follows of reference No. 22.
4. Edited the correct symbol.
5. Added the references that indicate the internal consistency of the questionnaire.

**Results:**

1. Cause of cancer pain is very complicated. In our clinical practice, we prescribed sustained and immediate-release opioids combined with (or without) other adjuvants analgesics in cancer pain treatment, for example, anticonvulsants, antidepressants, Non-steroidal Anti-inflammatory drugs, or acetaminophen. 
2. In the section, the patient factors in the part of knowledge score of the questionnaire. A pain specialist created the knowledge assessment of strong opioid analgesics that were completely mentioned in the method section.
3. In the part of face-to-face in-depth interviews by the co-authors. The interviews were audio-recorded, and the interviewers also took field notes. The study was conducted until data saturation was reached that approved by primary and secondary authors.
4. A result of medication-related factors, the patients mentioned using the rescue opioids for sleep well rather than for analgesic effects.

**Discussion:**

1. The authors elaborate on the study results so that it is clear in the description of the second theme (ineffective pain control). Some patients increased their basal opioid doses because their pain had worsened and dared not to call to consult the doctor about ineffective pain control by many reasons. Among the interviews, some patients expressed thoughtfulness.
2. In our study, the patients mentioned as following the results in theme 1: Poor understanding and non-acceptance of the disease.
3. In table 3. Univariate and multivariate logistic regression results. In our study, more than three types of pain medication had significant associations with opioid non-adherence (P = 0.032), which related to the quantitative study that the patients mentioned about adverse effects and multiple organ failure if using medications in the long-term.

# Peer review: Reviewer response for version 1

**DOI:** 10.5256/f1000research.30656.r76209

**Published:** 2020-12-23

**Authors:** Sasikaan Nimmaanrat

**Affiliations:** 1 Songklanagarind Hospital, Faculty of Medicine, Prince of Songkla University, Hat Yai, Thailand; 2 Department of Anesthesiology, Faculty of Medicine, Prince of Songkla University, Hat Yai, Thailand

This study used a mixed method of a survey and an in-depth interview to evaluate factors affecting opioid non-adherence in cancer patients. The questionnaire survey was to identify adherence and non-adherence patients, followed by an in-depth interview in patients with non-adherence until data were saturated. Using the mixed method makes the study more informative and attractive. The flow of the study was clearly presented. 

 Adequate pain management in cancer patients is mandatory. It is useful to know which factors lead to poor pain control. The authors have found factors which can be classified into patient factors, medication-related factors and illness-related factors. Knowing the contributing factors for inadequate cancer pain management can lead to strategic planning to modify these factors resulting in better pain relief.

Is the work clearly and accurately presented and does it cite the current literature?

Yes

If applicable, is the statistical analysis and its interpretation appropriate?

Yes

Are all the source data underlying the results available to ensure full reproducibility?

Yes

Is the study design appropriate and is the work technically sound?

Yes

Are the conclusions drawn adequately supported by the results?

Yes

Are sufficient details of methods and analysis provided to allow replication by others?

Yes

Reviewer Expertise:

Pain management including acute, cancer and chronic non-cancer pain.

I confirm that I have read this submission and believe that I have an appropriate level of expertise to confirm that it is of an acceptable scientific standard.

# Response

**Published:** 2020-12-28

**Authors:** Rattaphol Seangrung

**Affiliations:** Ramathibodi Hospital, Thailand

Thank you very much.
